# Establishment and characterization of METON myoepithelioma cell line derived from human palatal myoepithelioma: apical reference to the diverse differentiation potential

**DOI:** 10.1007/s13577-013-0066-7

**Published:** 2013-06-13

**Authors:** Minako Suzuki, Hiroshi Ishikawa, Miyuki Kawakami, Taka Nakahara, Akira Tanaka, Izumi Mataga

**Affiliations:** 1 Department of Oral and Maxillofacial Surgery, School of Life Dentistry at Niigata, The Nippon Dental University, Niigata, Japan; 2 Division of Cell Regeneration and Transplantation, Advanced Research Center, School of Life Dentistry at Niigata, The Nippon Dental University, Niigata, Japan; 3 Department of NDU Life Sciences, School of Life Dentistry at Tokyo, The Nippon Dental University, Niigata, Japan; 4 Department of Developmental and Regenerative Dentistry, School of Life Dentistry at Tokyo, The Nippon Dental University, Niigata, Japan; 5 Department of Oral and Maxillofacial Surgery, Niigata Hospital, The Nippon Dental University, Niigata, Japan

**Keywords:** Myoepithelioma, Cell line, Progenitor cells, Salivary gland tumors, Subclones

## Abstract

Myoepithelioma is an extremely rare condition that accounts for 1–1.5 % of salivary gland tumors. It was formerly regarded as a subtype of pleomorphic adenoma, in which myoepithelial structural components predominated, but was listed as a separate disease entity in the 1991 World Health Organization classification (Seifert in Histological typing of salivary gland tumours. Springer, Berlin, 1991). Its histology is highly varied and recurrence is frequent (El-Naggar et al. in J Larygol Otol 103:1192–1197, 1989), with cases of malignant transformation having been reported (Seifert in Histological typing of salivary gland tumours. Springer, Berlin, 1991; Barnes et al. in Pathology and Genetics of head and neck tumours. IARC Press, Lyon, 2005), making this a difficult tumor to control in many cases. This is thought to be due to the multiple differentiation potential of myoepithelial cells, but the details are unknown. There have been a number of reports of the establishment of cell lines (Shirasuna et al. Cancer. 45:297–305, 1980; Jaeger et al. Oral Surg Oral Med Oral Pathol Oral Radiol Endod 84:663–667, 1997), but numerous points remain unclear. We established a myoepithelial cell line designated METON, and investigated its characteristics. METON consists of cells with two different morphologies: spindle-shaped cells and epithelial-like cells. Then. we also used single-cell cloning method to establish various subclones (epithelial-like, spindle-like, and mixed epithelial-like/spindle-like cell lines). Among these, pluripotency markers were expressed by the mixed epithelial-like/spindle-like cell lines. The newly established cell line expressing these pluripotency markers will be extremely useful for elucidating the diverse histologies of salivary gland tumors.

## Introduction

Myoepithelioma was first described in 1972 by Sheldon et al. [6], and its classification was changed from pleomorphic adenoma to a separate disease in the 1991 World Health Organization (WHO) classification [1]. It is an extremely rare condition that accounts for 1–1.5 % of salivary gland tumors [3, 7]. It commonly occurs between the ages of 20 and 69 years, irrespective of sex. It is most frequently observed in the parotid glands, followed by the palatine glands [1]. Recently, cases of myoepithelioma arising in the skin and deep soft tissue have also been reported [8–15]. The chief structural component of this tumor is tumorous epithelium, but it exhibits a diverse histology in which the epithelium and mesenchyme are mixed. This is thought to be due to the multiple differentiation potential of myoepithelial cells, but the details are unknown. Clinically, recurrence is frequent [2], with cases of malignant transformation having been reported [1, 3], making this a difficult tumor to control in many cases.

Myoepithelial cells are derived from ectoderm and are contractile, combining the properties of epithelium with those of smooth muscle. They are found in the tissue of exocrine glands such as salivary, mammary, sweat, prostate, and lacrimal glands, where they are involved in the secretion and transport of secretions [16]. Myoepithelioma represents the tumorous transformation of these myoepithelial cells, and is characterized by a large number of histological subtypes, with the proportions of these different subtypes reported as epithelial type (45.0 %), spindle type (32.5 %), hyaline type (7.5 %), clear type (2.5 %), and mixed type (12.5 %) [3, 17].

We have succeeded in establishing a cell line from an epithelial-type myoepithelioma, a benign salivary gland tumor that possesses these specific properties, and here report on its characteristics.

## Materials and methods

### Personal history

**Fig. 1:**
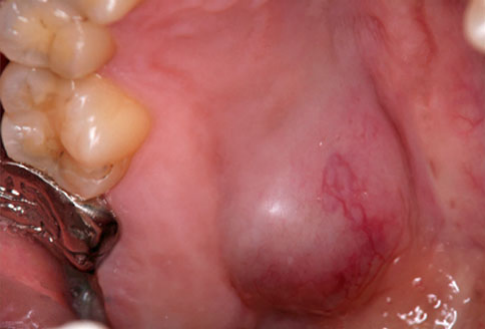
Personal history intraoral photograph: a well-demarcated, elastic-soft, painless tumor 12 × 15 mm in size was evident in the right palate. There was some surface reddening

**Fig. 2:**
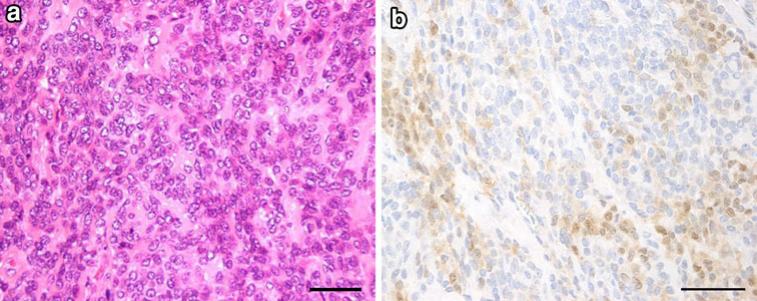
Histopathology and immunostaining of resected tissue. **a** The tumor was well demarcated by fibrous connective tissue, with solid proliferation of polygonal tumor cells inside the tumor. It was diagnosed as epithelial-type myoepithelioma. **b** Immunostaining for S-100 protein. Immunostaining was positive for S-100 protein. *Bars* 50 μm

The patient was a 30-year-old woman who noticed a swelling in her right palate in September 2010 and was examined in our department on November 16, 2010. On initial examination, a well-demarcated, elastic-soft, painless tumor 12 × 15 mm in size was evident in the right palate, with some surface reddening (Fig. 1). CT revealed pressure resorption of the palatal bone on the right side, but there was no continuity with the nasal cavity or the maxillary sinus. On MRI, the region corresponding to the tumor in the right palate was around the same intensity as muscle in T1-weighted imaging, with heterogeneous high intensity evident on T2-weighted imaging. The palatal tumor was completely removed under general anesthesia on February 8, 2011, and a portion of the resected tumor was transferred to growth medium at 4 °C and transported to the laboratory. Hematoxylin and eosin staining of the resected tissue led to a pathological diagnosis of epithelial-type myoepithelioma (Fig. 2).

### Culture materials and methods

After removal, tumor tissue was immediately placed in growth medium (GM) [(DMEM/F12; Life Technologies, Grand Island, NY, USA) supplemented with 15 % fetal bovine serum (FBS) (Batch:G121-6; JRS, Woodland, CA, USA), 0.1 % non-essential amino acids solution (Life Technologies), 100 mM/ml GlutaMAX (Life Technologies), 50 U/ml penicillin and 50 μg/ml streptomycin (Life Technologies) and 0.25 μg/ml Fungizone (Life Technologies)] and was then kept at 0 °C within the culture room [18]. Whole manipulations in the cell culture (primary culture, subculture and cryopreservation) were performed using 5-ml disposable pipettes (VWR Serological Pipettes, Westchester, PA, USA). The tumor was rinsed several times with Hanks’ solution (Nissui, Tokyo, Japan) supplemented with the previously described antibiotics (same concentrations). The tumor mass was cut into small pieces using razor blades. All of the fragments were collected and placed into a 15-ml (Greiner Plastics, Frickenhausen, Bavaria, Germany) and/or 50-ml centrifugal tubes (Falcon Plastics, Franklin Lakes, NJ, USA) with GM. Tubes were then centrifuged at 300*g* for 5 min. The sediment was resuspended with GM, followed by culture with GM in 60-mm dishes (Falcon Plastics). In the primary cultures, three kinds of cells, epithelial-like cells, spindle-like cells, and fibroblastic cells, were observed. The fibroblastic cells disappeared gradually during cultivation, and METON cell line was established. For cryopreservation, cultures were removed using 0.2 % trypsin-0.02 % EDTA/PBS(-) solution (Trypsin 250; Difco, Detroit, MI, USA) and Hanks’ solution at room temperature. After centrifugation (300*g*, for 5 min), cells were dispersed in GM supplemented with 10 % DMSO (about 2 × 10^6^ cells/1.8 ml).

### Cell culture observations

During cell culture, cells were observed and photographed using an inverted phase-contrast microscope (IX 71; Olympus, Tokyo, Japan).

### Electron microscopic observations

For electron micrography, cells in the 35-mm dishes were fixed using 2.5 % glutaraldehyde in 0.1 M phosphate buffer for 1 h at room temperature, and were then post-fixed with 1 % OsO_4_ in the same buffer at 0 °C for 30 min. Cells were rinsed with the same buffer, dehydrated with ethanol, immersed twice in absolute propylene oxide, and embedded in Quetol 812. Sections were cut at a thickness of 90–100 nm with a diamond knife and mounted onto grids. Following staining with uranyl acetate and lead citrate, cells were observed with a JEOL JEM-1200 EX-II electron microscope at 80 kV.

### Chromosome analysis

Direct chromosome preparation was performed during the exponential growth phase of the cells at passage 5. Cells at 90 % confluence were incubated with 1 × 10^−7^ M colcemid (Sigma Aldrich, MI, USA) for 60 min at 37 °C. Cells were then harvested and centrifuged. Cells were resuspended in 70 mM KCl at a concentration of 1 × 10^4^ cells/ml. After incubating for 20 min at 37 °C, cells were centrifuged and fixed with freshly prepared fixative (methanol:acetic acid, 3:1). After cells were centrifuged, the supernatant was decanted. The pellet was left overnight at 4 °C before being resuspended in the 3:1 methanol:acetic acid fixative. The cell suspension was dropped onto a wet, cold microglass slide and then stained with Giemsa solution. A total of 50 mitotic figures were randomly chosen, analyzed and then counted for distribution and karyotype.

### Xenotransplantation

Approximately 2 × 10^7^ cells/0.5 ml of Hanks’ solution per mouse were harvested using a syringe with a 23-gauge needle. Cells were then transplanted into the subcutis of the back of the neck of three nude mice (female BALB/cA, aged 5 weeks; KREA, Tokyo, Japan).

### Immunohistochemical staining

For immunohistochemical staining of the cells, Laboratory-Tek II chamber slides (Cat. 154534; Nalge Nunc, Roskilde, Denmark) were used. Cells were stained with anti-human actin monoclonal antibody (1:500) (produced in our laboratory), anti-human myosin polyclonal antibody (1:1,000) (produced in our laboratory), anti-human S-100 monoclonal antibody (1:400) (Sigma Aldrich), anti-human cytokeratin polyclonal antibody (1:1,000) (Daco, Glostrup, Denmark).

### Single cell cloning

Using growth medium, we prepared a cell suspension in which the number of cells was diluted to a density of 1 cell/ml, and divided the suspension at 200 μl in each well of a 96-well multi-well plate. Under a phase-contrast microscope, we marked wells containing single cells, and separated the cell groups grown from single cells as subclones [19].

### RNA sampling and RT-PCR RNA

Sampling of total RNA from petri dishes containing cells was performed using the RNeasy Mini Kit^®^ (QIAGEN, Hilden, Germany), in accordance with the manufacturer’s protocol. cDNA synthesis was performed with the High Capacity cDNA Reverse Transcription Kit (Applied Biosystems Japan, Tokyo, Japan), using 1 μg of total RNA. PCR amplification was performed using the Platinum PCR Super Mix (Life Technologies) with gene-specific primers for SOX2, Nanog, Oct3/4 and PSCA. PCR cycling conditions were as follows: 35 cycles of denaturation at 94 °C for 30 s, annealing at 55 °C for 30 s, and extension at 72 °C for 60 s. GAPDH was used as the internal standard under the same PCR amplification conditions. PCR products underwent electrophoresis with 2 % agarose gel (Nippon Gene, Tokyo, Japan) and bands were visualized with ethidium bromide.

## Results

### Phase-contrast micrograph (Fig. 3)

During the initial stage of culture, METON contained numerous spindle-shaped cells, but epithelial-like cells gradually increased as culture continued. These epithelial-like cells were scattered in conglomerations within the spindle-shaped cells. METON consisted of cells of these two morphological types.

**Fig. 3:**
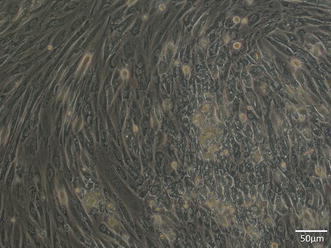
Phase-contrast micrography. The cell line consisted of cells of two morphological types, epithelial-like and spindle-shaped

### Transmission electron microscopy (Fig. 4)

Cells had spherical or elliptical nuclei, and well-defined nucleoli were present. They contained well-developed microfilaments, mitochondria and lysosomes. Numerous desmosomes were evident between the epithelial-like cells, but myofibrils rather than desmosomes were present between the spindle-shaped cells.

**Fig. 4:**
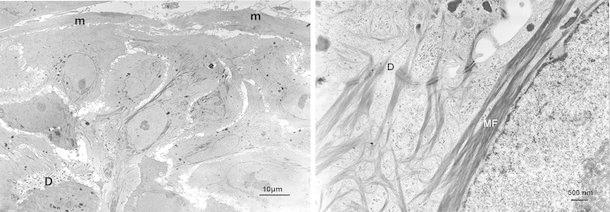
Transmission electron micrograph. Both cell types contained nuclei with well-defined nucleoli. Well-developed microfilaments (*MF*), mitochondria (*m*), lysosomes, and other structures were evident within cells. Cells were bonded here and there with desmosomes (*D*)

### Karyotypic analysis (Fig. 5)

Chromosome mode was 46, and 88 % of cells in this cell line were normal diploid cells.

**Fig. 5:**
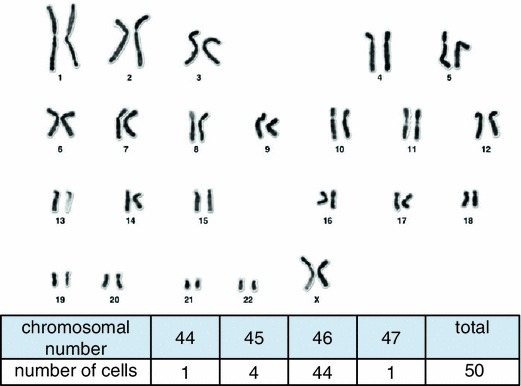
Karyotype. Chromosome mode was 46, and 88 % of cells in this cell line were normal diploid cells

### Fluorescence immunostaining (Fig. 6)

Immunostaining was positive for the skeletal muscle markers α-SMA and myosin as well as the epithelium-specific marker cytokeratin. Although fluorescence immunostaining was not positive for S-100, its expression was observed on RT-PCR.

**Fig. 6:**
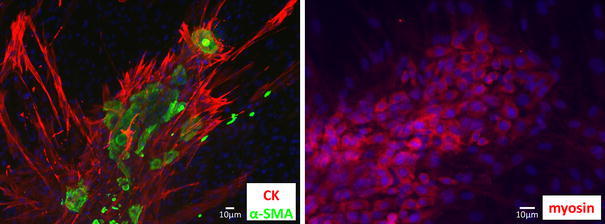
Fluorescence immunostaining. Nuclear staining was seen for 4′6′-diamidino-2-phenylindole dihydrochloride (DAPI). *Bars* 10 μm

### Single cell cloning (Fig. 7)

In order to investigate the diverse differentiation potential of myoepithelioma, we used single-cell cloning method to isolate epithelial-like cells and spindle-shaped cells, and three kinds of subclones, epithelial-like, spindle-like, and mixed epithelial-like/spindle-like morphologies, were established successfully from single cells.

**Fig. 7:**
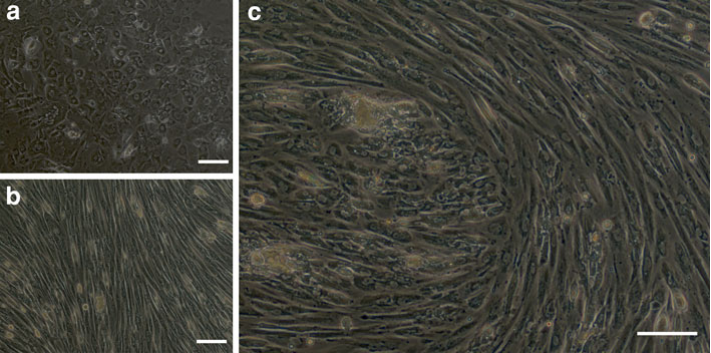
Phase-contrast micrograph of single-cell lines. **a**. Epithelial-like subclones established from single cell (8 subclones). **b** Spindle-like subclones established from single cell (10 subclones). **c** Mixed epithelial-like/spindle-like subclones established from single cell (11 subclones). *Bars* 50 μm

### RT-PCR (Fig. 8)

The fact that mixed epithelial-like/spindle-like cell lines were generated by single-cell cloning, despite the fact that they were cloned from single cells, suggested that myoepitheliomas contain undifferentiated cells. We therefore used RT-PCR to test for pluripotency markers, and confirmed the presence of undifferentiated cells by the expression of the salivary gland pluripotency marker PSCA in METON and of the proliferation markers Oct3/4 and PSCA in the mixed epithelial-like/spindle-like cell lines. No pluripotency marker expression was evident in the epithelial-like or spindle-like cell lines.

**Fig. 8:**
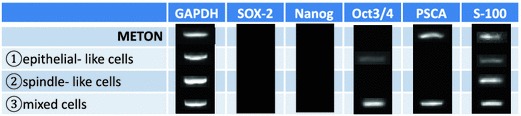
RT-PCR of subclones (epithelial-like, spindle-like, and mixed epithelial-like/spindle-like cell lines) dissociated from METON

## Discussion

We succeeded in establishing a cell line from a myoepithelioma that arose in the palate of a 30-year-old woman. The METON cell line we established consisted of cells of two morphological types: epithelial-like and spindle-like. We carried out single-cell cloning to establish subclones in order to determine whether these cell types are independent cells or whether they transform into one another, as well as to investigate the presence of progenitor cells, and found that cell lines were generated that contained both epithelial-like and spindle-like cells despite being derived from a single cell. This suggests that progenitor cells capable of differentiating into both epithelial-like and spindle-like cells were present. Histological differentiation diversity may thus contribute to the process of myoepithelioma formation.

The difference between myoepithelioma and pleomorphic adenoma is the subject of frequent debate. They both generally consist of fibrous, hyaline, chondromatous and myxomatous mesenchymal components, and tumorous myoepithelial cells [20]. There is no great difference between their structures, with the main point being whether duct formation is evident. According to the WHO classification and Ellis et al. [7], there is no ductal differentiation in pleomorphic adenoma and it lacks cartilage-like myxomatous stroma, whereas Dardick [17, 21] stated that myoepithelioma should be diagnosed if the proportion of duct formation was less than 5–10 % of the entire tumor. Salivary gland tumors such as myoepithelioma and pleomorphic adenoma thus have very complex histological structures with diverse differentiation. For this reason, and because there have been reports of their possessing tissue infiltrative potential or undergoing malignant transformation [1, 3], some authors believe that resection should also include healthy tissue during their treatment by surgical removal [22].

According to the 1991 WHO classification, positive immunohistochemical staining for S-100 protein and either actin or myosin is proof of myoepithelial cells. In fact, however, it is rare for tumorous myoepithelium to exhibit differentiation similar to that of normal myoepithelium, and as these tests may be negative in some cases, it has come to be accepted that variation exists. According to the new 2005 WHO classification [16], because the frequency and intensity of positive staining vary by cell type, a combination of cytokeratin and the smooth muscle markers vimentin, S-100 protein, and glial fibrillary acidic protein (GFAP) should also be used to assist diagnosis. We found that immunostaining of the resected tumor was positive for α-SMA (not shown) and S-100 protein. Immunostaining of METON showed that the spindle-shaped cells were positive for muscle markers such as α-SMA and myosin, and the epithelial-like cells were positive for cytokeratin. Although cytoimmunofluorescence was negative for S-100 protein, its expression was observed on RT-PCR. S-100 protein immunostaining of the resected tumor also showed that expression by positive cells differed according to location, with variation also present in the numbers of positive cells. This suggests that cells that synthesize little S-100 protein predominated in our culture system.

RT-PCR screening for pluripotency markers in the three types of subclone line obtained by single-cell cloning revealed the expression of the salivary gland pluripotency marker PSCA in the myoepithelioma cell lines and the mixed epithelial-like/spindle-like cell lines, confirming the presence of undifferentiated cells.

We also screened for Sox-2, Nanog, and Oct3/4, which are transcription factor markers expressed in pluripotent cells. Sox-2 has been reported to be involved in the function of pluripotency, and Oct3/4 and Nanog in its maintenance [23]. Only PSCA was observed in the myoepithelioma cell lines, with no expression of Sox-2, Nanog, or Oct3/4. However, Oct3/4 expression was present in the mixed epithelial-like/spindle-like cell lines. This supports the assumption that each subclone consisted of cells with the potential to differentiate into epithelial-like and spindle-like cells, despite being derived from a single cell. It also suggests that the presence of such cells among tumor cells in myoepithelioma, and their contribution to the maintenance of salivary gland differentiation potential, may be one cause of the complexity of their histological structure and their diverse differentiation.

Our results suggested that the presence of undifferentiated cells is one factor in the great variety of histological types characteristic of myoepithelioma, and that these undifferentiated cells (with the potential to differentiate into either epithelial-like or spindle-like cells) may be the main cellular component of this tumor with proliferative potential. The cell line with differentiation potential that we have established provides an extremely useful experimental model for elucidating matters such as the starting point for the formation of salivary gland tumors with diverse histologies.
